# The Forgotten Complication of Recurrent Nephrolithiasis: “Squamous Cell Carcinoma of the Renal Pelvis”—A Case and Review of the Literature

**DOI:** 10.1155/2015/298317

**Published:** 2015-10-22

**Authors:** Tarek Assi, Elie El Rassy, Samah Naderi, Tania Moussa, Maroun Moukarzel, Joseph Kattan

**Affiliations:** ^1^Department of Oncology, Hotel Dieu de France University Hospital, Faculty of Medicine, Saint Joseph University, Beirut 1104 2020, Lebanon; ^2^Pathology Department, Hotel Dieu de France University Hospital, Faculty of Medicine, Saint Joseph University, Beirut 1104 2020, Lebanon; ^3^Radiology Department, Hotel Dieu de France University Hospital, Faculty of Medicine, Saint Joseph University, Beirut 1104 2020, Lebanon; ^4^Department of Urology, Hotel Dieu de France University Hospital, Faculty of Medicine, Saint Joseph University, Beirut 1104 2020, Lebanon

## Abstract

Squamous cell carcinoma (SCC) of the renal pelvis is an aggressive tumor with insidious onset of unspecific symptoms and advanced stages at diagnosis. It is a rare entity, accounting for 0.5–8% of renal tumors. In this paper, we describe the case of a patient with a history of recurrent nephrolithiasis that presented with an aggressive form of SCC of the renal pelvis with rapid relapse after resection.

## 1. Introduction

Squamous cell carcinoma (SCC) of the renal pelvis is rare tumor accounting for 0.5–8% of renal tumors [1]. This tumour has an insidious onset of unspecific symptoms. It is highly aggressive and often detected at an advanced stage. Few cases of SCC of the renal pelvis have been described in the literature. Therefore little is known about the optimal management of this type of tumor. In this paper, we present the rare case of a patient, with a history of multiple surgeries for nephrolithiasis, diagnosed with an aggressive form of SCC of the renal pelvis.

## 2. Case Report

**Figure 1:**
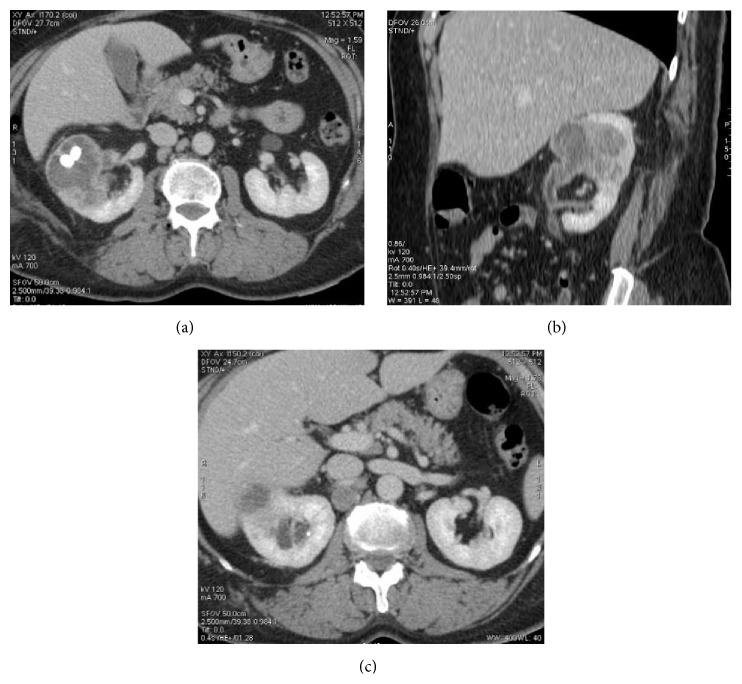
Preoperative CT scan. (a) Axial and (b) coronal enhanced CT scan showing a mixed solid and cystic mass at the middle third of the right kidney containing two centimetric renal calculi. (c) Axial enhanced CT scan showing a centimetric lymph node in the retrocaval region.

**Figure 2:**
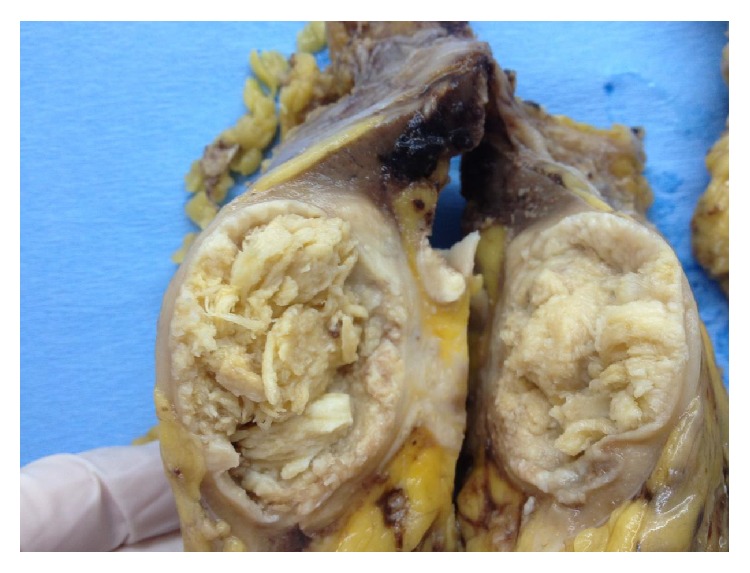
Gross examination showing a well-circumscribed, light tan to yellow mass in mid pole of left kidney, measuring 4.2 cm × 4 cm.

**Figure 3:**
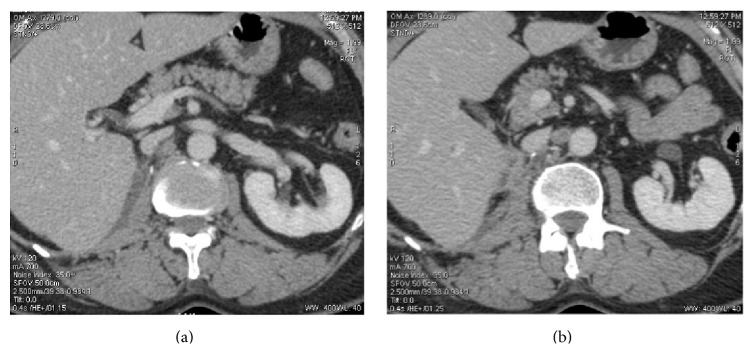
Postoperative CT scan. Axial enhanced CT scan showing (a) tissue thickening of 9 mm between the inferior vena cava and the right diaphragmatic pillar and (b) recent appearance of centimetric interaortocaval lymph node.

**Figure 4:**
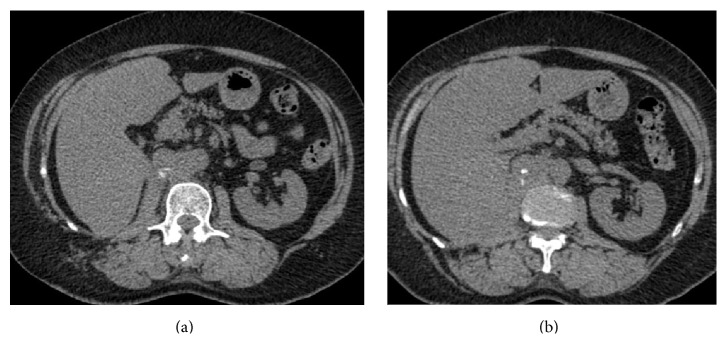
CT scan performed after 4 cycles of chemotherapy. Axial nonenhanced CT scan showing (a) increase in size of the interaortocaval lymph node now measuring 2.5 cm indistinguishable from the aorta and the vena cava; (b) increase in retrohepatic tissue thickening along the vena cava.

An otherwise healthy 61-year-old female presented to our clinic with a previous medical history of nephrolithiasis requiring repetitive percutaneous nephrolithotomy that occurred more than 10 years ago. The patient was incompliant to regular urological follow-up. Upon her most recent presentation, she complained of right flank pain with persistent macrohematuria that started one month ago. Abdominal palpation revealed tenderness on right lower quadrant as well as costovertebral tenderness. No abnormalities were noted on routine blood and urine tests. Investigation by an abdominal CT scan revealed an obstructing cortical mass, with mixed solid and cystic components, at the middle third level of the right kidney. The lesion measured 4.5 cm and contained two calculi of 1 cm each with few centimetric lymph nodes along the para-aortic region (Figure 1). No distant metastases were detected on subsequent thoracic CT scan. The patient underwent laparoscopic-assisted right nephrectomy without any complications. On gross examination, the mid pole of the kidney was occupied by a partially cystic mass measuring 4.2 × 4 cm, infiltrating the renal pelvic wall, the renal parenchyma, and the renal sinus fat. No macroscopic extension into perinephric tissue was observed (Figure 2). Microscopic examination of the tumor submitted in toto revealed a moderately differentiated SCC with marked keratinization. Renal sinus fat and renal sinus vein invasion were identified. Renal capsule, vessels, and perinephric fat were free of tumor. There was no evidence of urothelial differentiation (invasive or in situ). Extensive squamous metaplasia of the urothelium in the renal pelvis was observed. Lymph node metastasis was found in two lateral caval lymph nodes, the largest measuring 3.5 cm (2/4), and in one hilar lymph node (1/1). A diagnosis of SCC of the renal pelvis (pT3N2M0) was made. Follow-up CT scan three weeks postoperatively noted a tissue thickening between the inferior vena cava and the right diaphragmatic pillar. Lymph nodes were detected along the abdominal aorta and the right primitive iliac artery (Figure 3). Consequently, the patient received four cycles of chemotherapy with Gemcitabine (1700 mg on Days 1 and 8) and Cisplatin (100 mg on Day 1 every 21 days). Subsequent CT scan performed after chemotherapy, without contrast injection due to moderate renal insufficiency, revealed progressive disease without resolution of affected lymph nodes (Figure 4). Unfortunately, progressive resistant disease precluded further surgical management and second-line treatment by Vinflunine (480 mg every 3 weeks) was started.

## 3. Discussion

Of all types of renal cancer, tumors of the upper urothelial tract represent only 5%. These tumors are most commonly transitional cell carcinomas [1, 2]. SCC is a rare entity in this location with unclear pathogenesis. It is thought that, under chronic stress, a predetermined pattern occurs over time with the development of squamous metaplasia, then progression to dysplasia and carcinoma [3]. In this setting, determining the presence of an urothelial dysplastic component classifies the tumor as urothelial carcinoma subtype [4]. Primary SCC of the renal pelvis is divided into peripheral and central SCC based on the location of the tumor, the latter being associated with poorer outcome due to higher rates of lymph node metastases [5].

Several stressors were reported to induce this malignant transformation and include nephrolithiasis, chronic urinary tract infection, renal tuberculosis, radiation therapy, percutaneous nephrostomy, immunosuppression, schistosomiasis, and vitamin A deficiency. Of these, the main risk factor almost constant in all reported cases of SCC of the renal pelvis is a history of long standing nephrolithiasis [6, 7]. Due to the rarity of the SCC of the renal pelvis and the high occurrence of kidney stones in the general population, clear recommendations for follow-up of patients with history of nephrolithiasis seem too difficult to achieve. High index of suspicion for serious complications among high risk patients with long-standing history of nephrolithiasis should always be maintained upon appearance or persistence of common urinary symptoms in order to detect SCC of the renal pelvis in early stages.

Clinical and radiological features of SCC of the renal pelvis are nonspecific. Patients commonly present in their sixth decade for paraneoplastic manifestations of hypercalcemia, leukocytosis, and thrombocytosis or hematuria and flank pain [7, 8]. The evaluation of the role of urine cytology in the diagnosis of upper urinary tract tumors has led to mixed results; although it has considerable sensitivity in detecting high-grade tumors, negative findings have been frequently found before the diagnosis of low-grade tumors [9]. Even though urinary cytology may show malignant cells, only 70% of positive results were correlated with the actual histopathological results so there is no consensus in the usage of this test in patients with high risk [10]. We did not perform urine cytology for our patient as the preplanned surgery would achieve a more accurate diagnosis. A CT finding of solid mass, hydronephrosis, or calcifications in association with renal calculi should be suspicious in high risk patients. The presence of enhancing extraluminal and exophytic mass are possible suggestive features [5]. The differential diagnosis includes xanthogranulomatous pyelonephritis and renal/extrarenal neoplasms [11]. SCC of the renal pelvis is often metastatic at the time of diagnosis and is associated with a poor prognosis (one-year survival is less than 10%) [5, 6].

Resection of the primary tumors remains the mainstay for treatment of SCC of the renal pelvis even in advanced and metastatic disease. Chemotherapy and concomitant chemoradiotherapy based treatment using Cisplatin, Bleomycin, and Methotrexate failed to show a significant amelioration in prognosis [6, 12]. In the case of a very early local relapse, as in our patient, immediate salvage surgery could not be of any benefit. The indication of chemotherapy for transitional cell carcinoma such as platinum and gemcitabine seems more adequate. No standard follow-up protocols are recommended for these patients but regular clinical examination, blood tests, and chest X-ray can be included for further evaluations.

In conclusion, SCC of the renal pelvis is a very rare and aggressive tumor. Locoregional relapse of the disease can occur early after the primary resection, as was seen in the present case. This highlights the need for early diagnosis in high risk patients. Complete surgical excision is necessary since this tumor is unresponsive to the available salvage therapies.
